# Structural insight into RNA encapsidation by the severe fever with thrombocytopenia syndrome virus nucleocapsid protein

**DOI:** 10.1128/mbio.02381-25

**Published:** 2025-10-31

**Authors:** Yong Wang, Hao Wu, Jiawen Sun, Wenhua Kuang, Hualin Wang, Yun-Jia Ning, Zengqin Deng

**Affiliations:** 1 State Key Laboratory of Virology and Biosafety, Wuhan Institute of Virology, Chinese Academy of Sciences 74614, Wuhan, Hubei, China; 2 University of Chinese Academy of Sciences 74519 , Beijing, China; 3 Hubei Jiangxia Laboratory, Wuhan, Hubei, China; Case Western Reserve University School of Medicine, Cleveland, Ohio, USA

**Keywords:** SFTSV, RNA encapsidation, Cryo-EM, nucleocapsid protein, drug targets

## Abstract

Severe fever with thrombocytopenia syndrome virus (SFTSV), an emerging highly pathogenic bunyavirus, poses a significant public health threat with a case fatality rate of up to 30%. The viral nucleocapsid protein (NP) encapsidates the genomic RNA to form a ribonucleoprotein (RNP) complex, which is critical for transcription and replication. However, the molecular mechanism underlying SFTSV RNA encapsidation remains poorly understood, largely due to the lack of structural information on the NP-RNA complex. Here, we report a cryo-electron microscopy structure of the SFTSV NP in complex with single-stranded RNA. The structure reveals a pentameric NP assembly that sequesters RNA along the inner surface of the oligomeric ring in a sequence-independent manner. Strikingly, all the RNA bases face the protein, rendering them inaccessible for transcription and replication. Each NP subunit accommodates four nucleotides within an evolutionarily conserved hydrophobic cleft, with an additional two to three nucleotides bound at the inter-subunit interface. The functional importance of the NP-RNA interactions is further corroborated by a minigenome-based assay. This work provides structural insight into RNA encapsidation by SFTSV NP and offers a foundation for the rational design of antiviral therapeutics targeting this essential viral protein.

**IMPORTANCE:**

Severe fever with thrombocytopenia syndrome virus (SFTSV) is a highly pathogenic bunyavirus that causes severe hemorrhagic fever, leukopenia, thrombocytopenia, and multi-organ failure, with a case fatality rate of up to 30%. No licensed vaccines or specific antiviral therapies are currently available. The viral nucleocapsid protein (NP) is essential for viral transcription and replication, forming a ribonucleoprotein complex (RNP) by encapsidating viral genomic RNA. However, the structural basis of RNA recognition and encapsidation by SFTSV NP remains poorly understood. In this study, we determined a cryo-electron microscopy structure of the SFTSV NP-RNA complex. Structural comparisons and evolutionary conservation analysis of NPs across the family Phenuiviridae uncovered a conserved RNA-binding mode among phenuiviruses, suggesting a shared RNA encapsidation mechanism among related viruses. Our findings provide critical structural insights into SFTSV RNA encapsidation and will aid future efforts to develop antivirals against SFTSV and related pathogenic viruses.

## INTRODUCTION

The class *Bunyaviricetes* is a large group of segmented negative-strand RNA viruses, encompassing numerous pathogenic viruses. Among them, the family *Phenuiviridae* includes two highly pathogenic human pathogens: severe fever with thrombocytopenia syndrome virus (SFTSV) and Rift Valley fever virus (RVFV) (1, 2). Currently, there are no licensed human vaccines or specific antiviral treatments available against SFTSV and RVFV, underscoring the urgent need for further research to develop effective countermeasures against these two highly pathogenic bunyaviruses. SFTSV, an emerging tick-borne virus, has rapidly become endemic in several East Asian countries, including South Korea, Japan, China, Vietnam, Pakistan, and Thailand, since its first identification in China in 2009 (3). Infection with SFTSV leads to severe hemorrhagic fever, leukopenia, thrombocytopenia, and multi-organ failure, with a case fatality rate of up to 30%. Although SFTSV infection remains primarily confined to Asia, its potential for global spread is a growing concern. The Asian longhorned tick (*Haemaphysalis longicornis*), the primary vector for SFTSV (4), has already been detected in the United States, Russia, Australia, and the Western Pacific (5–7), raising alarms about SFTSV’s possible emergence beyond its current endemic regions.

Like other bunyaviruses, SFTSV contains three single-stranded RNA genome segments designated as large (L), medium (M), and small (S). The L segment encodes an RNA-dependent RNA polymerase, also referred to as the L protein, that is responsible for viral genome transcription and replication. The M segment encodes the glycoproteins Gn and Gc, which form heterodimers covering the viral surface and facilitate cellular attachment, entry, and fusion. The S segment encodes the nucleocapsid protein (NP) that encapsidates the viral genomic RNA to form the ribonucleoprotein (RNP) complex. The S segment also encodes an important virulence factor, nonstructural protein (NSs), via an ambisense coding strategy. RNP plays critical roles in protecting the viral RNA from host RNase degradation and serving as functional templates of viral polymerase to synthesize viral mRNA and genome RNA.

Crystallographic studies revealed that the wild-type SFTSV NP forms pentameric and hexameric rings, while a SFTSV NP mutant forms a tetramer, mediated by the flexible N-terminal arm (8, 9). The C-terminal core harbors a putative RNA-binding cleft on the inner surface of the oligomeric ring. Notably, the inhibitor suramin binds to this putative RNA-binding site and suppresses viral replication (8), underscoring the potential for developing therapeutic strategies targeting this viral protein. RNA co-purified with recombinant SFTSV NP is resistant to degradation by external nuclease, further supporting NP’s protective role (9). Despite these advances in the biochemical and structural characterization of SFTSV NP, key questions remain unresolved, including the detailed architecture of the NP-RNA complex, the specificity and stoichiometry of RNA binding, and the mechanistic basis of RNP assembly.

In this study, we determined the cryo-electron microscopy (cryo-EM) structure of the SFTSV NP-RNA complex. By combining structural analysis, structure-guided mutagenesis, and functional assays, we provide insights into RNA recognition and encapsidation, offering a foundation for rational antiviral design targeting SFTSV NP.

## RESULTS

### Recombinant SFTSV NP oligomerizes and binds cellular RNA

**Fig 1:**
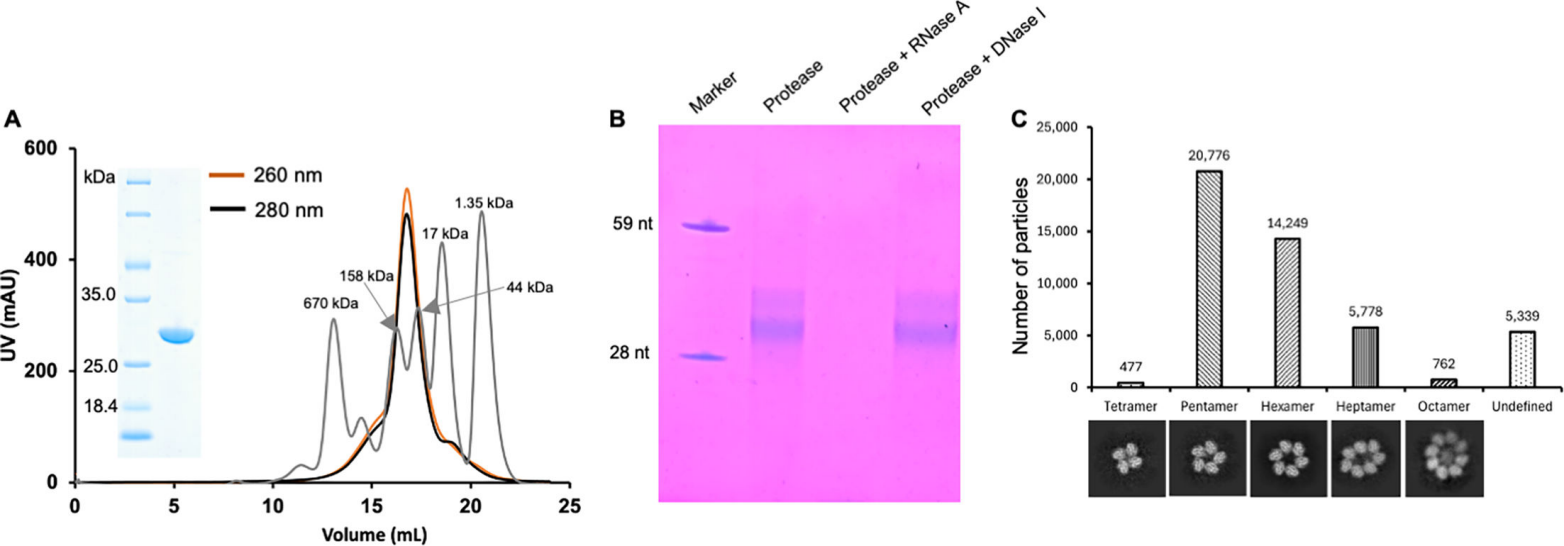
Purification and characterization of the SFTSV NP-RNA complex. (**A**) SEC of the *E. coli*-expressed SFTSV NP. The absorbance at 260 and 280 nm of SFTSV NP is indicated in orange and black, respectively. The gel filtration profile of the standard proteins (Biorad Gel Filtration Standard #1511901) is colored gray. Coomassie-stained denaturing gel (inset) shows the high purity of the purified SFTSV NP protein. (**B**) RNase A digestion of the purified SFTSV NP indicates the presence of RNA. (**C**) Cryo-EM analysis of the purified SFTSV NP. After two rounds of reference-free 2D classification, a total of 47,381 protein particles were selected for analysis. The distribution of oligomeric states is summarized, and the representative 2D classification images of each oligomeric state are shown.

We successfully expressed the full-length SFTSV NP in *Escherichia coli*. During purification by size-exclusion chromatography (SEC), the protein was eluted as a major peak at a retention volume of ~16.9 mL, corresponding to a molecular weight exceeding 100 kDa. SDS-PAGE analysis confirmed that the peak fractions contain a highly pure protein with the expected size for SFTSV NP (27 kDa) (Fig. 1A). These results suggest that the recombinant SFTSV NP forms a higher-order oligomer in solution, consistent with previous studies (8, 9). Moreover, the ratio of the optical density (OD) at 260 nm to the OD at 280 nm of the major protein peak is ~1.1, indicating that the purified SFTSV NP encapsidates nucleic acids. Denaturing urea PAGE after protease treatment resolved two major nucleic acid bands with molecular weight slightly larger than a 28-nt single-stranded RNA (ssRNA). These nucleic acids were completely degraded upon RNase A treatment but remained unaffected by DNase I treatment, indicating that the recombinant SFTSV NP predominantly binds RNA (Fig. 1B).

A previous negative-staining study reported heterogeneity in the oligomeric state of the recombinant SFTSV NP in solution, with assemblies ranging from tetramers to hexamers (9). To further elucidate its oligomerization, we performed cryo-EM analysis. From 550 micrographs, we selected 193,082 particles for 2D classification. After two rounds of reference-free 2D classification, 47,381 particles were retained for analysis. These particles exhibited a predominant pentameric (20,776 particles, 43.8%) and hexameric (14,249 particles, 30.0%) organization, with minor populations of heptamers and trace amounts of tetramer and octamer (Fig. 1C; Fig. S1).

### Structure determination of the SFTSV NP-RNA complex

Initial attempts to solve the cryo-EM structure of the SFTSV NP-RNA complex were unsuccessful due to the extreme preferred orientation of the sample (Fig. S1). To address this issue, we treated the sample with methyl-PEG4-NHS ester to PEGylate the protein (10). The PEGylated sample showed diverse views after 2D classification, allowing for further 3D reconstruction (Fig. S2A and B). Despite five distinct oligomeric states of the recombinant SFTSV NP existing in solution, likely due to preferred orientation issues of the other states, we were only able to determine the cryo-EM structure of the pentameric assembly at an overall resolution of 3.98 Å (Fig. S2B). Notably, imposing C5 symmetry during data processing did not improve the resolution and density quality of the reconstruction, indicating intrinsic conformational flexibility among protomers within the NP pentamer. Local resolution analysis revealed that the NP-RNA interaction interfaces were better resolved (around 3 Å) than the solvent-exposed regions, likely due to their higher structural rigidity. Using the crystal structure of SFTSV NP as an initial model, we docked and refined the model against the cryo-EM density. The density corresponding to the co-purified RNA was clearly visualized, allowing unambiguous tracing of a 28-nucleotide single-stranded RNA bound to the NP pentamer (Fig. S2F). A poly(U) RNA sequence was arbitrarily modeled within the complex structure. The final refined model has good geometry and fits well into the electron density map (Table S1).

### Overall structure of the SFTSV NP-RNA complex

**Fig 2:**
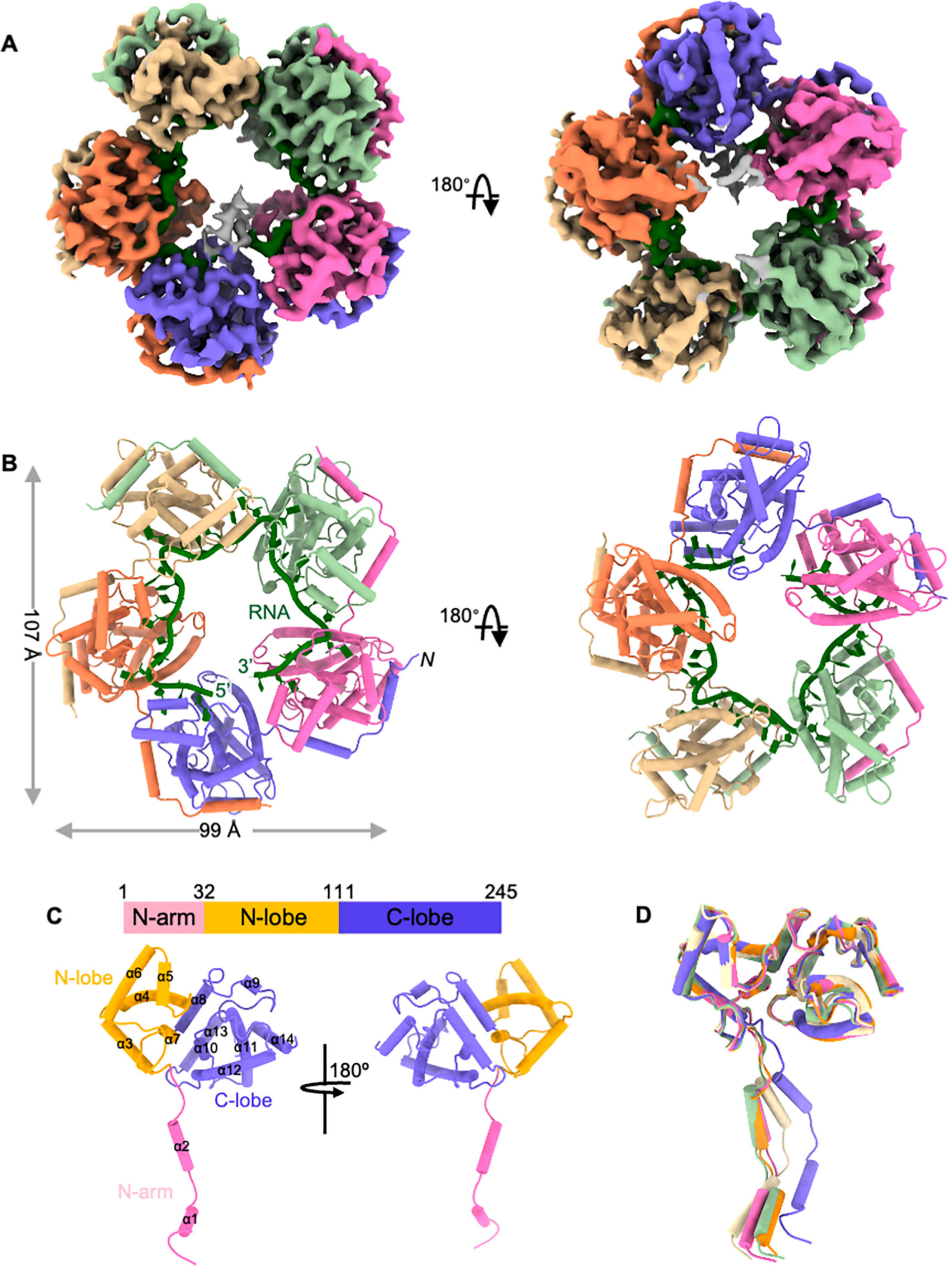
Cryo-EM structure of the SFTSV NP-RNA complex. (**A**) Cryo-EM reconstruction of the SFTSV NP-RNA complex. Each subunit of the pentameric NP is in a different color: the ssRNA is colored in forest green. (**B**) Overall structure of the SFTSV NP-RNA complex. Each subunit and ssRNA follows the same color scheme as in Fig. 1A. (**C**) Structure of a single NP subunit. Each domain is labeled and in a different color. (**D**) Superimposition of five NP subunits shows the conformational changes of the N-arm.

**Fig 3:**
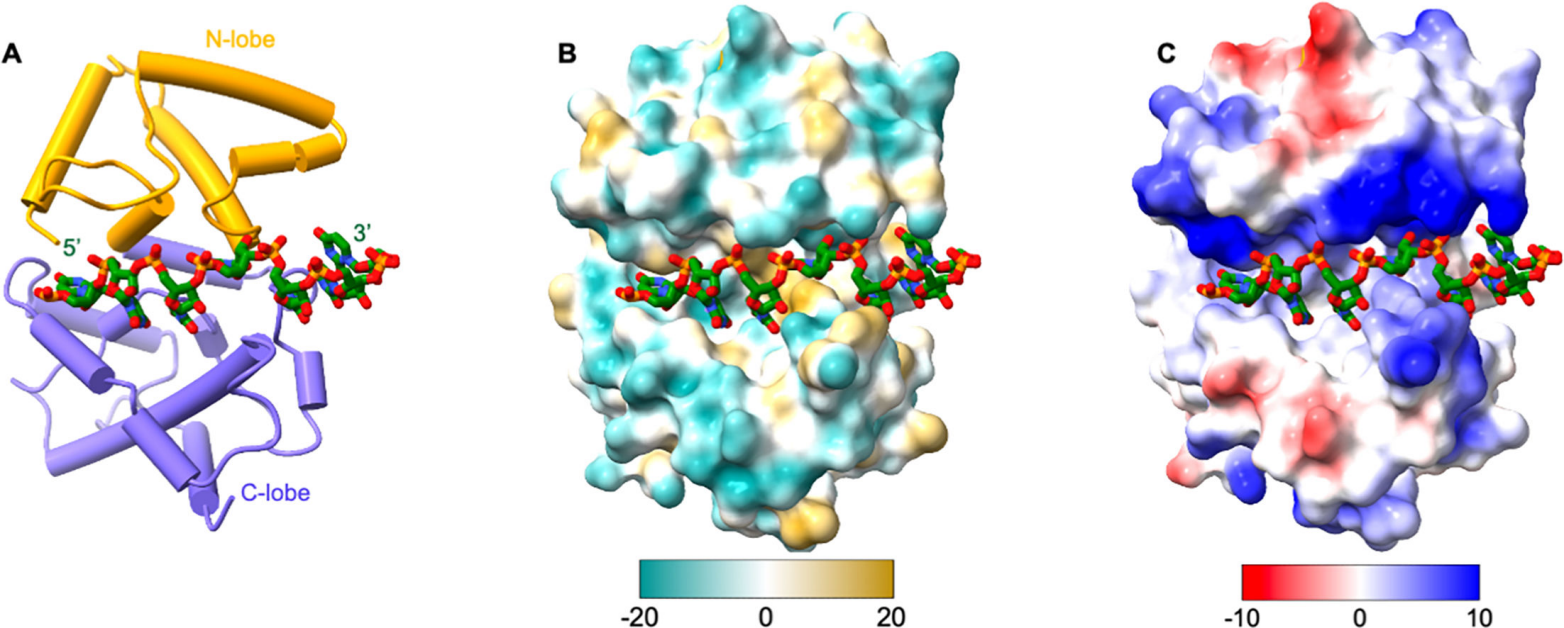
RNA binding by the SFTSV NP. ssRNA is bound in the cleft formed by N-lobe and C-lobe, with NP shown as a cartoon (**A**), surface colored by hydrophobicity (**B**), or surface colored by electrostatic potential (**C**).

The pentameric SFTSV NP-RNA complex adopts a ring-like architecture with overall dimensions of 99 Å × 107 Å ×60 Å. Within this structure, the resolved 28-nt ssRNA is bound along the inner circumference of the ring (Fig. 2A and B). Each NP subunit consists of three distinct regions: the N-terminal arm region (N-arm), the middle N-lobe, and the C-terminal C-lobe (Fig. 2C). The middle N-lobe and C-terminal C-lobe together form the subunit core. While the subunit cores exhibit only minor conformational variations among protomers, the N-arm displays significant positional flexibility relative to the core, with an all-atom root-mean-square deviation (RMSD) ranging from 2.7 to 3.6 Å across different subunits (Fig. 2D). The N-arm inserts into the surface groove of the adjacent subunit, forming extensive arm-to-core interactions that mediate NP oligomerization. Each NP subunit contains a deep, narrow RNA-binding cleft that collectively forms a continuous groove along the inner surface of the pentameric ring. This groove features a hydrophobic interior lined with multiple positively charged residues at its rim. RNA binds with the bases inserted into the cleft and the sugar-phosphate backbone oriented toward the center of the ring (Fig. 3). Detailed analysis reveals two distinct RNA-binding stoichiometries per NP subunit: each can accommodate either 6 or 7 nucleotides (Fig. S3A). Specifically, four nucleotides are bound within the primary RNA-binding cleft, while an additional 2–3 nucleotides are located at the subunit interface (Fig. S3B and C). Thus, the number of nucleotides accommodated at the subunit interface determines whether an NP protomer binds 6 or 7 nucleotides in total. In the complex structure, we can unambiguously resolve the nucleotides at the subunit interface for four protomers, revealing an alternating pattern of 6 and 7 nucleotides around the pentamer. Structural comparison with the RNA-free SFTSV NP core structure demonstrates that RNA binding induces a conformational transition from a closed to an open state, involving slight rotations of both the N-lobe and C-lobe (Fig. S3D).

### Detailed interactions between SFTSV NP and RNA

**Fig 4:**
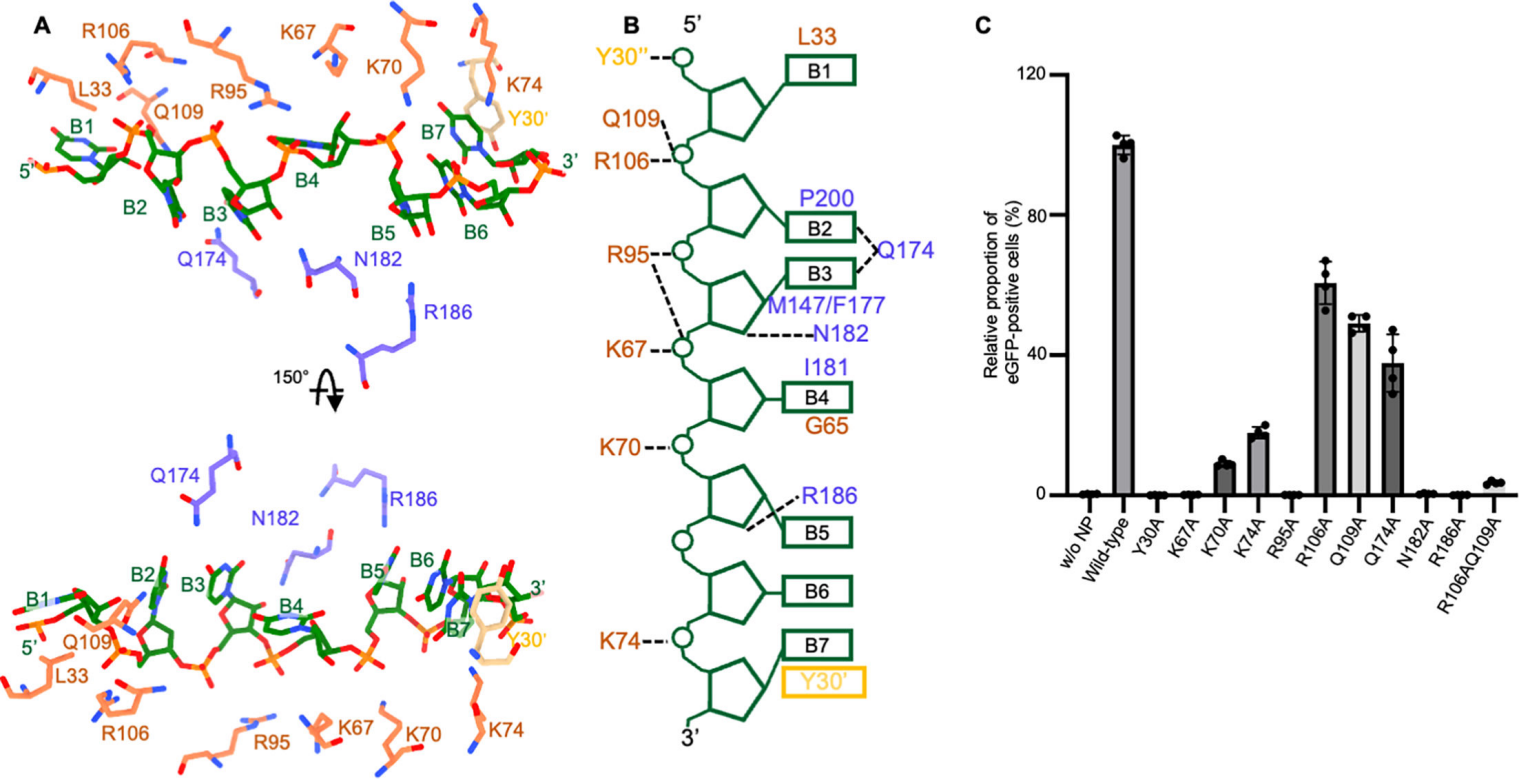
Detailed interactions between RNA and SFTSV NP. (**A**) Detailed interactions between RNA and SFTSV NP, only residues involved in polar and base-stacking interactions are shown. Residues from the N-lobe or the C-lobe are colored salmon and medium slate blue, respectively. One residue, Y30 from the neighboring subunit, is colored wheat. (**B**) Schematic drawing highlighting the SFTSV NP-RNA interactions. The RNA and interacting residues are colored consistently with panel A. (**C**) Effect of substitution of the RNA-interacting residues on the SFTSV minigenome activity in the enhanced green fluorescent protein (eGFP) reporter system (in standardized relative light units relative to the wild-type SFTSV NP). Data are presented as mean ± SD, *n* = 4. Representative images of minigenome assays are shown in Fig. S4.

The RNA segments (6 or 7 nt) bound to each NP subunit exhibit similar conformations, with the primary difference occurring at the 3′ terminus. In the 7-nt segment, three bases are stacked at this position, whereas only two are stacked in the 6-nt segment (Fig. S3B and C). Here, we focus on the detailed interactions observed in the 7-nt binding mode (Fig. 4A and B). In each NP subunit, the 5′-most base (B1) is stacked against residue L33. The B2 and B3 bases are stacked within the central compartment of the RNA-binding cleft, which is lined with the side chains of hydrophobic residues M147, F177, I181, F197, and P200. Base B4 inserts into a hydrophobic pocket formed by residues G65, V105, P127, M147, F177, and I181. The three 3′-most bases (B5-7) form a stacking interaction, with B7 stacking with residue Y30 from a neighboring subunit. In addition to hydrophobic and base-stacking interactions, NP forms a network of polar contacts with the RNA 5′ phosphates (P1 to P7) of all nucleotides except P6: residue Y30 from a neighboring subunit with P1; R106 and Q109 with P2; R95 with P3 and P4; K67 with P4; K70 with P5; K74 with P7. Furthermore, Q174 forms hydrogen bonds with RNA bases 2 and 3. Residues N182 and R186 form hydrogen bonds with the 2′-OH of nucleotide 3 and nucleotide 5, respectively. The majority of the interactions involve polar contacts with the RNA backbone and hydrophobic interactions with the nucleobase, suggesting a sequence-independent RNA-binding mode.

To corroborate our structural findings, we examined the function of NP mutants using a cell-based minigenome system. An M RNA analog containing the M untranslated regions (UTRs) and a negative-sense enhanced green fluorescent protein (eGFP) gene sequence was subcloned into a pRF42 vector under the control of pol I promoter to allow the generation of a viral genome-like RNA segment. After co-transfection with the NP and L genes under the control of the CAG promoter into cells, NP recognizes the RNA UTRs and assembles with the minigenome into functional RNPs, then replication and transcription by the L protein can occur, resulting in reporter gene expression. To disrupt RNA-binding interactions, polar residues implicated in RNA binding were substituted with alanine. Mutating most basic residues (lysine and arginine), as well as residue Y30, abolished the eGFP signal. Interestingly, alanine substitution of residues N182 and R186, which recognize 2′-OH group of RNA, also completely abolished the expression of eGFP. By contrast, mutating residue Q174 had only a moderate effect. Both residues R106 and Q109 interact with the 5′ phosphate of nucleotide 5. While a single mutation of either residue had a limited impact, the double mutation significantly reduced eGFP expression (Fig. 4C and Fig. S4). These results underscore the functional significance of the identified RNA-interacting residues of SFTSV NP.

### Structural convergence and divergence of bunyavirus NP-RNA complexes

**Fig 5:**
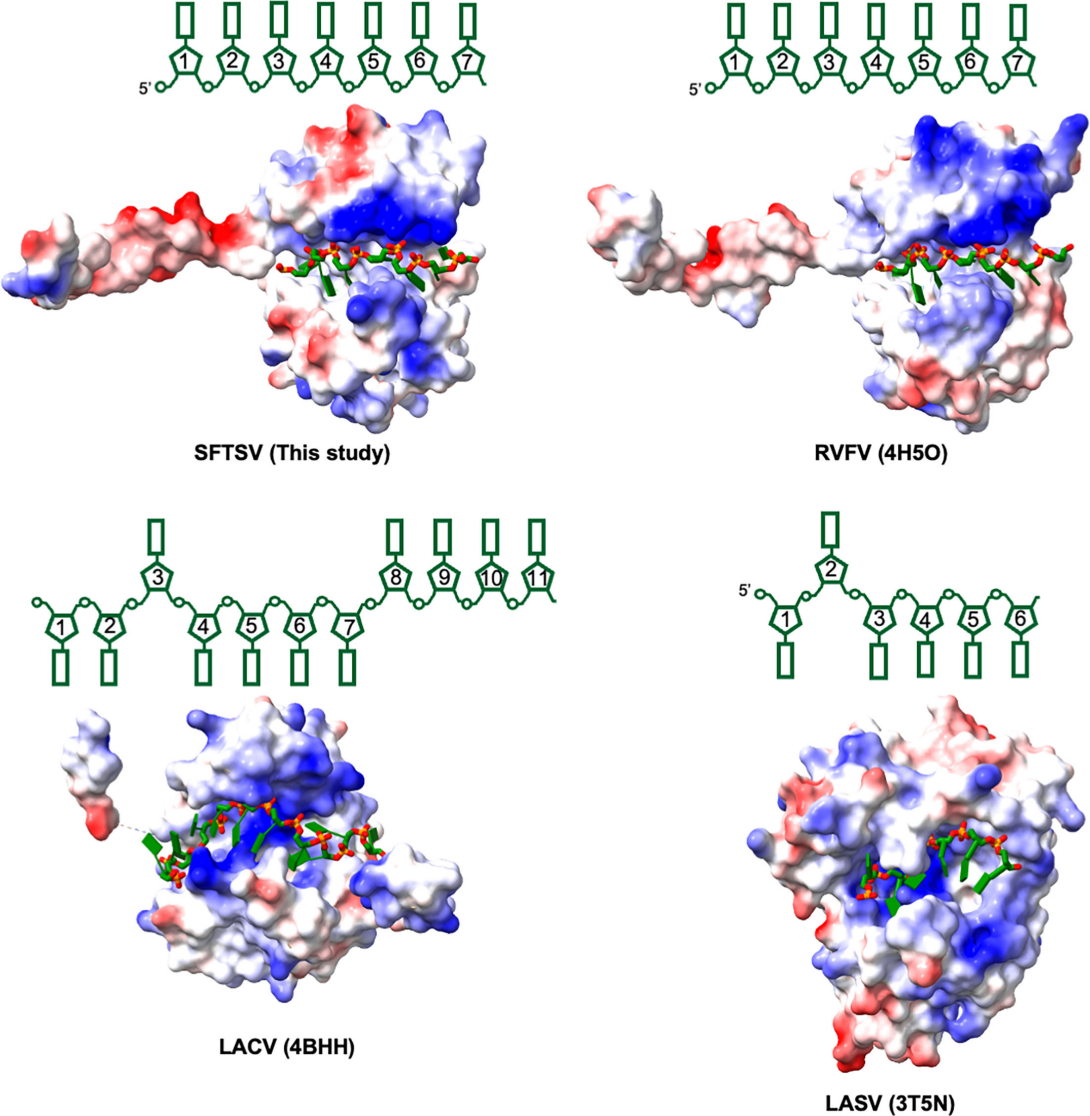
Structural comparison of the RNA-binding mode of bunyavirus NP-RNA complexes. Electrostatic potential maps illustrate the RNA-binding interface, with RNA represented in a stick model. Corresponding schematic diagrams (above each map) depict the bound RNA in the complexes. Bases oriented toward the top indicate inward-facing interactions with the protein, while those oriented toward the bottom suggest the bases are solvent-exposed. The PDB ID of each structure is shown in parentheses.

Structures of the NP-RNA complexes have been reported for several bunyaviruses, including RVFV, Toscana virus, La Crosse Virus (LACV), Leanyer virus (LEAV), Schmallenberg virus (SBV), Bunyamwera viruses (BUNV), Lassa fever virus (LASV), Hantaan virus (HTNV), and Tomato spotted wilt virus (TSMV) (11–19). All of these viral NPs form oligomers in complex with ssRNA, except for LASV NP. The LASV NP alone forms a trimer with N- and C-terminal domains arranged in a head-to-tail manner (20), while the N-terminal domain of the LASV NP forms a complex with ssRNA as a monomer. The NP-RNA complexes of HTNV and TSMV bind only short RNA fragments with their sugar-phosphate backbones oriented toward a positively charged cleft. By contrast, RVFV, Toscana virus, and SFTSV, all members of the family *Phenuiviridae*, exhibit an RNA-binding mode where RNA bases are sequestered in a deep hydrophobic groove in a sequence-independent manner (Fig. 5). Structural superposition of the NP subunit of SFTSV NP-RNA with those of RVFV and Toscana virus highlights their high structural similarity, yielding RMSDs of 1.3 Å over 208 and 199 aligned Cα atoms per NP subunit, respectively (Fig. S5A). Additionally, evolutionary conservation analysis of 154 phenuiviral NPs using the consurf server indicates that the RNA-binding cleft is the most conserved region, formed by highly conserved residues (21) (Fig. S5B). These findings suggest that NPs within the *Phenuiviridae* family may share a highly conserved RNA-binding mode.

LACV, BUNV, LEAV, and SBV, all belonging to the *Orthobunyavirus* genus, adopt a similar mode for RNA sequestration. For example, each LACV NP subunit binds 11 nucleotides, with U3 and U8-11 positioned at the base of the RNA-binding cleft, facing inward, while the remaining bases remain solvent-exposed (Fig. 5). In contrast, LASV (a member of *Arenaviridae*) exhibits a distinct RNA-binding mode. Each LASV NP binds six nucleotides with the sugar-phosphate backbone directed into an RNA-binding pocket. Five of six bases are oriented outward and fully exposed to the solvent, a striking difference compared to SFTSV and LACV (Fig. 5). These findings underscore that viruses within the same genus or family might employ a conserved structural fold and manner for genome encapsidation, whereas NPs from different genera or families exhibit significant divergence in RNA-binding mechanisms.

## DISCUSSION

The SFTSV NP-RNA complex structure, determined in this study, and the RVFV and Toscana virus NP-RNA complexes, along with the evolutionary conservation analysis of NPs across the family *Phenuiviridae*, potentially provide a clue to the mechanism of viral genome packaging. In each NP subunit, four RNA bases are deeply embedded within a conserved hydrophobic cleft, while an additional two to three bases at the subunit interface remain protein-facing. This extensive sequestration sterically prevents RNA bases from Watson-Crick pairing, rendering the encapsidated RNA inaccessible for transcription or replication unless actively remodeled by the viral polymerase or unidentified factors. The remarkable stability of these NP-RNA interactions further explains why obtaining RNA-free NPs of SFTSV, RVFV, and Toscana virus requires either denaturing conditions or extensive nuclease digestion (8, 9, 19, 22).

The genomic RNA of bunyaviruses is encapsidated by NP to form RNPs, which not only facilitate viral RNA replication and transcription but also protect the viral genome from degradation by host RNases. In addition to these essential roles, NPs from certain bunyaviruses contribute to suppression of the host innate immune response and mediate RNP packaging into virus-like particles through interactions with the viral envelope glycoproteins (23–25). Despite the conserved functional role, NP structures exhibit remarkable diversity across different bunyavirus families. Structural studies have revealed that the recombinant NP-RNA complexes from various bunyaviruses typically form oligomers *in vitro*. However, whether these oligomeric states represent the functional RNP organization in authentic virions remains unclear. RNPs of RVFV and another phenuivirus, Uukuniemi virus, isolated from infected cells display a string-like morphology that lacks higher-order symmetry (22, 26). The measured width of these authentic virus RNPs suggests that monomeric NP-RNA units, rather than the resolved NP or NP-RNA oligomers, serve as the fundamental building blocks (11, 27). Given the similar NP-NP and highly conserved NP-RNA interactions, we hypothesize that SFTSV assembles its RNP through a mechanism analogous to that of RVFV.

In the cryo-EM map, an unmodeled density connects the 5′ and 3′ ends of the RNA, likely corresponding to unmodeled terminal regions (Fig. S6A). If these terminal sequences are complementary, they could form an RNA duplex, a scenario well documented in bunyaviruses. The 5′ and 3′ termini of each genomic segment are highly conserved within a given bunyavirus species and exhibit strong complementarity, enabling the formation of a duplex stem (28). This interaction allows each segment to potentially adopt a circularized panhandle structure. We propose a model in which SFTSV RNPs adopt a circular organization facilitated by both RNA panhandles and NP-NP interactions (Fig. S6B). The viral RNA polymerase recognizes the panhandle structure to initiate RNA synthesis. During elongation, the genomic RNA, which is encapsidated by the NPs near the replication center, becomes transiently uncoated, making it accessible to the RNA polymerase. Meanwhile, the NPs remain held in position by the flexible N-arm. After the RNA polymerase passes through, the temporarily dislodged genomic RNA is re-encapsidated by the NPs.

The evolutionarily conserved hydrophobic RNA-binding cleft in the NP represents a promising drug target for developing broad-spectrum therapeutics against medically important phenuiviruses. Indeed, the inhibitor suramin binds within this conserved groove with micromolar affinity and effectively suppresses SFTSV replication (Fig. S7). Notably, suramin also exhibits comparable binding affinity to NPs from other *Phenuiviridae* members, including RVFV, Buenaventura virus, and Granada virus, but shows no detectable binding to NPs from orthobunyavirus, nairovirus, or hantavirus (8). Given the striking structural conservation of this RNA-binding pocket in phenuiviruses, further investigation is warranted to determine whether suramin exhibits antiviral activity against additional phenuiviruses by targeting NPs.

In summary, our SFTSV NP-RNA complex structure reveals a conserved RNA sequestration mechanism within the family *Phenuiviridae*, advancing our understanding of viral RNA encapsidation. These findings provide a foundation for developing antivirals against SFTSV, RVFV, and related pathogenic viruses.

## MATERIALS AND METHODS

### Protein expression and purification

DNA sequence encoding SFTSV NP (MN510206.2) was cloned into a pET28a vector containing a C-terminal His tag and transformed into *E. coli* BL21(DE3) cells. Protein expression was induced by the addition of isopropyl β-D-thiogalactopyranoside to a final concentration of 0.4  mM at 16°C when the OD_600_ reached 0.6–0.8, and the cells were cultured for another 16 h. Cells were harvested via centrifugation at 5,000 × *g* for 10 min at 4°C. The collected cells were then resuspended in lysis buffer (20  mM Tris-HCl, pH 8.0, 150 mM NaCl) with protease inhibitor (1  mM PMSF) and lysed using a high-pressure cell crusher (Union-Biotech) at 600 bar. The resulting lysate was centrifuged at 30,000 × *g* for 60 min at 4°C. The supernatant was collected, loaded onto Ni-charged Resin FF (GenScript), and washed with 50 mL of wash buffer (20 mM Tris-HCl, pH 8.0, 150 mM NaCl, and 100 mM imidazole). The target protein was eluted with the elution buffer containing 20 mM Tris-HCl, pH 8.0, 150 mM NaCl, and 500 mM imidazole. Subsequent purification was performed using a Superose 6 Increase 10/300 Gl SEC column (Cytiva) pre-equilibrated with buffer containing 20 mM Tris-HCl, pH 8.0, and 150  mM NaCl. Peak fractions containing the SFSTV NP were pooled and concentrated to ~10 mg/mL and stored at −80°C for future use.

### Denaturing polyacrylamide gel electrophoresis

To identify the type of nucleic acids co-purified with SFTSV NP, 20 µL of SFTSV NP (10 mg/mL) was treated with proteinase K at a final concentration of 0.1 mg/mL and incubated at 37°C for 30 min. The mixture was then divided equally into two aliquots: one was treated with DNase I (20 µg/mL), and the other with RNase A (20 µg/mL), followed by incubation at 37°C for another 30 min. Loading buffer (50% glycerol) was added to each sample, which was then denatured at 95°C for 5 min. The samples were resolved on a 12% (wt/vol) polyacrylamide gel containing 7 M urea and stained with Stains-all. Two ssRNA markers were included as references (28-nt ssRNA: CUGUGCUCUUUUUUUCACAGUUUUUGAU, 59-nt ssRNA: GCUUUAUCAGAAGCCAGACAUUAACGCUUCUGGAGAAACUCAACGAGCUGGACGCGGAU). The gel was visualized using a gel imaging system, ChemiDoc Imaging System (Biorad).

### PEGylation of SFTSV NP

SFTSV NP was diluted to 1–2 mg/mL in 20 mM HEPES-NaOH, pH 8.0, and 150 mM NaCl, then incubated with methyl-PEG4-NHS ester (PEG4) at a final concentration of 4 mM for 30 min at 37°C. The reaction was quenched by adding 1/10 (vol/vol) 1 M Tris-HCl, pH 8.0, and the proteins were further purified using a Superose 6 Increase 10/300 Gl column (Cytiva) to remove excess PEG4. Peak fractions containing the PEGylated SFTSV NP were pooled and concentrated to ~5  mg/mL and stored at −80°C for future use.

### Cryo-EM sample preparation

Purified protein (3.5 µL) at a concentration of 0.6 mg/mL (SFTSV NP) or 2.4  mg/mL (PEGylated SFTSV NP) was applied to the glow-discharged Cu 200 mesh R1.2/1.3 holey carbon grids (Quantifoil). After a 20 s incubation, the grids were blotted for 2 s with a blot force of 0 at 100% humidity and 4°C, then plunge-frozen using Vitrobot Mark IV (FEI, Thermo Fisher Scientific).

### Cryo-EM data collection and image processing

Cryo-EM data were collected with a CRYO ARM 300 electron microscope (JEOL, Japan) operating at 300 kV, with a K3 direct electron detector (Gatan, United States). Data were collected at a nominal magnification of 50,000× in a super-resolution counting mode, with a pixel size of 0.475 Å/pixel. Movies were automatically collected using Serial-EM software (29) at a frame rate of 40 frames per second, accumulating a total dose of 40 e/Å^2^ within a defocus range of −0.5 to −2.5 µm. Patch-based motion correction and CTF estimation of the recorded movies were performed in cryoSPARC v4.2.0 (30). Particles were automatically picked by Topaz picking from 4850 micrographs and extracted with a particle box size of 240 pixels and subsequently subjected to 2D classification. In all, 788,291 particles from good classes were selected for further heterogeneous refinement with six initial ab initio models generated by *ab initio* reconstruction in cryoSPARC. One good class was selected for subsequent 3D classification, requesting three classes. One class containing 63,930 particles was selected for further NU-refinement, yielding a map with an overall resolution of 3.98  Å.

### Model building and refinement

Crystal structure of the SFTSV NP in RNA-free (PDB: 4J4U) was placed into the cryo-EM density map using UCSF Chimera. The RNA was manually built as poly(U) to fit the densities. Cycles of model building in COOT (31) and refinement using real_space_refine in Phenix (32) were performed to obtain the final refined model. The quality of the final models was analyzed with MolProbity in Phenix (33). Refinement statistics are summarized in Table S1.

### SFTSV minigenome reporter assay

SFTSV minigenome reporter assay was conducted as previously described (34, 35). Briefly, BHK21 cells seeded in 96-well plates were co-transfected with a PolI-MUTR-eGFP transcription plasmid producing the M RNA analog containing M UTRs and a negative-sense eGFP gene sequence, together with the SFTSV L protein expression plasmid and the plasmids encoding SFTSV wild-type NP (as positive control) or one of the NP mutants, or the empty vector (as negative control), using Lipofectamine 3000 transfection reagent (Invitrogen, Cat#L3000015). At 48 h post-transfection, cells were fixed with 4% paraformaldehyde, permeabilized with 0.5% Triton X-100, and blocked with 5% BSA in PBS. To visualize the expression of wild-type NP or mutants, cells were incubated with an anti-SFTSV NP polyclonal antibody at 4℃ overnight, followed by staining with Alexa Fluor 647-conjugated anti-rabbit IgG antibody (Abcam, Cat#ab150077) for 1 h at room temperature. To visualize the nuclei, cells were stained with Hoechst33258 (Beyotime, Cat#C1011) for 15 min at room temperature. Imaging and quantification of eGFP-positive and total cells per well were performed using the Operetta CLSTM high-throughput system (PerkinElmer). Relative minigenome reporter activity (%) = [(number of eGFP-positive cells of sample group)/(number of total cells of sample group)] ÷ [(number of eGFP-positive cells of positive control group)/(number of total cells of positive control group)] × 100.

## Data Availability

The cryo-EM maps have been deposited in the Electron Microscopy Data Bank with accession codes EMD-64333. Atomic coordinates have been deposited to the Protein Data Bank (PDB) with accession code 9UMZ.

## SUPPLEMENTAL MATERIAL

Supplemental material mbio.02381-25-s0001.pdf Fig. S1-S7 and Table S1.

ASM does not own the copyrights to Supplemental Material that may be linked to, or accessed through, an article. The authors have granted ASM a non-exclusive, world-wide license to publish the Supplemental Material files. Please contact the corresponding author directly for reuse.

## References

[1] Casel MA, Park SJ, Choi YK. 2021. Severe fever with thrombocytopenia syndrome virus: emerging novel phlebovirus and their control strategy. Exp Mol Med 53:713–722. doi:10.1038/s12276-021-00610-1 33953322 PMC8178303

[2] Gibson S, Noronha LE, Tubbs H, Cohnstaedt LW, Wilson WC, Mire C, Mitzel D, Anyamba A, Rostal M, Linthicum KJ. 2023. The increasing threat of Rift Valley fever virus globalization: strategic guidance for protection and preparation. J Med Entomol 60:1197–1213. doi:10.1093/jme/tjad113 37862067

[3] Yu X-J, Liang M-F, Zhang S-Y, Liu Y, Li J-D, Sun Y-L, Zhang L, Zhang Q-F, Popov VL, Li C, et al.. 2011. Fever with thrombocytopenia associated with a novel bunyavirus in China. N Engl J Med 364:1523–1532. doi:10.1056/NEJMoa1010095 21410387 PMC3113718

[4] Zhang X, Zhao C, Cheng C, Zhang G, Yu T, Lawrence K, Li H, Sun J, Yang Z, Ye L, et al.. 2022. Rapid spread of severe fever with thrombocytopenia syndrome virus by parthenogenetic asian longhorned ticks. Emerg Infect Dis 28:363–372. doi:10.3201/eid2802.211532 35075994 PMC8798674

[5] Egizi A, Bulaga-Seraphin L, Alt E, Bajwa WI, Bernick J, Bickerton M, Campbell SR, Connally N, Doi K, Falco RC, et al.. 2020. First glimpse into the origin and spread of the Asian longhorned tick, Haemaphysalis longicornis, in the United States. Zoonoses Public Health 67:637–650. doi:10.1111/zph.12743 32638553

[6] Heath ACG. 2020. A history of the introduction, establishment, dispersal and management of *Haemaphysalis longicornis* Neumann, 1901 (Ixodida: Ixodidae) in New Zealand. N Zealand J Zool 47:241–271. doi:10.1080/03014223.2020.1772326

[7] Miao D, Dai K, Zhao GP, Li XL, Shi WQ, Zhang JS, Yang Y, Liu W, Fang LQ. 2020. Mapping the global potential transmission hotspots for severe fever with thrombocytopenia syndrome by machine learning methods. Emerg Microbes Infect 9:817–826. doi:10.1080/22221751.2020.1748521 32212956 PMC7241453

[8] Jiao L, Ouyang S, Liang M, Niu F, Shaw N, Wu W, Ding W, Jin C, Peng Y, Zhu Y, Zhang F, Wang T, Li C, Zuo X, Luan CH, Li D, Liu ZJ. 2013. Structure of severe fever with thrombocytopenia syndrome virus nucleocapsid protein in complex with suramin reveals therapeutic potential. J Virol 87:6829–6839. doi:10.1128/JVI.00672-13 23576501 PMC3676114

[9] Zhou H, Sun Y, Wang Y, Liu M, Liu C, Wang W, Liu X, Li L, Deng F, Wang H, Guo Y, Lou Z. 2013. The nucleoprotein of severe fever with thrombocytopenia syndrome virus processes a stable hexameric ring to facilitate RNA encapsidation. Protein Cell 4:445–455. doi:10.1007/s13238-013-3901-4 23702688 PMC4875558

[10] Zhang Z, Shigematsu H, Shimizu T, Ohto U. 2021. Improving particle quality in cryo-EM analysis using a PEGylation method. Structure 29:1192–1199. doi:10.1016/j.str.2021.05.004 34048698

[11] Raymond DD, Piper ME, Gerrard SR, Skiniotis G, Smith JL. 2012. Phleboviruses encapsidate their genomes by sequestering RNA bases. Proc Natl Acad Sci USA 109:19208–19213. doi:10.1073/pnas.1213553109 23129612 PMC3511139

[12] Reguera J, Malet H, Weber F, Cusack S. 2013. Structural basis for encapsidation of genomic RNA by La Crosse orthobunyavirus nucleoprotein. Proc Natl Acad Sci USA 110:7246–7251. doi:10.1073/pnas.1302298110 23589854 PMC3645531

[13] Niu F, Shaw N, Wang YE, Jiao L, Ding W, Li X, Zhu P, Upur H, Ouyang S, Cheng G, Liu Z-J. 2013. Structure of the Leanyer orthobunyavirus nucleoprotein–RNA complex reveals unique architecture for RNA encapsidation. Proc Natl Acad Sci USA 110:9054–9059. doi:10.1073/pnas.1300035110 23569220 PMC3670306

[14] Hastie KM, Liu T, Li S, King LB, Ngo N, Zandonatti MA, Woods VL, de la Torre JC, Saphire EO. 2011. Crystal structure of the Lassa virus nucleoprotein–RNA complex reveals a gating mechanism for RNA binding. Proc Natl Acad Sci USA 108:19365–19370. doi:10.1073/pnas.1108515108 22084115 PMC3228486

[15] Arragain B, Reguera J, Desfosses A, Gutsche I, Schoehn G, Malet H. 2019. High resolution cryo-EM structure of the helical RNA-bound Hantaan virus nucleocapsid reveals its assembly mechanisms. elife 8:e43075. doi:10.7554/eLife.43075 30638449 PMC6365055

[16] Komoda K, Narita M, Yamashita K, Tanaka I, Yao M. 2017. Asymmetric trimeric ring structure of the nucleocapsid protein of tospovirus. J Virol 91:e01002-17. doi:10.1128/JVI.01002-17 28768868 PMC5625519

[17] Dong H, Li P, Böttcher B, Elliott RM, Dong C. 2013. Crystal structure of Schmallenberg orthobunyavirus nucleoprotein-RNA complex reveals a novel RNA sequestration mechanism. RNA 19:1129–1136. doi:10.1261/rna.039057.113 23798666 PMC3708532

[18] Li B, Wang Q, Pan X, Fernández de Castro I, Sun Y, Guo Y, Tao X, Risco C, Sui S-F, Lou Z. 2013. Bunyamwera virus possesses a distinct nucleocapsid protein to facilitate genome encapsidation. Proc Natl Acad Sci USA 110:9048–9053. doi:10.1073/pnas.1222552110 23569257 PMC3670369

[19] Olal D, Dick A, Woods VL Jr, Liu T, Li S, Devignot S, Weber F, Saphire EO, Daumke O. 2014. Structural insights into RNA encapsidation and helical assembly of the Toscana virus nucleoprotein. Nucleic Acids Res 42:6025–6037. doi:10.1093/nar/gku229 24688060 PMC4027202

[20] Qi X, Lan S, Wang W, Schelde LM, Dong H, Wallat GD, Ly H, Liang Y, Dong C. 2010. Cap binding and immune evasion revealed by Lassa nucleoprotein structure. Nature 468:779–783. doi:10.1038/nature09605 21085117 PMC3057469

[21] Yariv B, Yariv E, Kessel A, Masrati G, Chorin AB, Martz E, Mayrose I, Pupko T, Ben-Tal N. 2023. Using evolutionary data to make sense of macromolecules with a “face-lifted” ConSurf. Protein Sci 32:e4582. doi:10.1002/pro.4582 36718848 PMC9942591

[22] Raymond DD, Piper ME, Gerrard SR, Smith JL. 2010. Structure of the Rift Valley fever virus nucleocapsid protein reveals another architecture for RNA encapsidation. Proc Natl Acad Sci USA 107:11769–11774. doi:10.1073/pnas.1001760107 20547879 PMC2900692

[23] Zhang WK, Yan JM, Chu M, Li B, Gu XL, Jiang ZZ, Li ZM, Liu PP, Yu TM, Zhou CM, Yu XJ. 2025. Bunyavirus SFTSV nucleoprotein exploits TUFM-mediated mitophagy to impair antiviral innate immunity. Autophagy 21:102–119. doi:10.1080/15548627.2024.2393067 39189526 PMC11702967

[24] Jiang X, Huang Q, Wang W, Dong H, Ly H, Liang Y, Dong C. 2013. Structures of arenaviral nucleoproteins with triphosphate dsRNA reveal a unique mechanism of immune suppression. J Biol Chem 288:16949–16959. doi:10.1074/jbc.M112.420521 23615902 PMC3675627

[25] Överby AK, Pettersson RF, Neve EPA. 2007. The glycoprotein cytoplasmic tail of Uukuniemi virus bunyaviridae interacts with ribonucleoproteins and is critical for genome packaging. J Virol 81:3198–3205. doi:10.1128/JVI.02655-06 17229712 PMC1866086

[26] Pettersson RF, von Bonsdorff CH. 1975. Ribonucleoproteins of Uukuniemi virus are circular. J Virol 15:386–392. doi:10.1128/JVI.15.2.386-392.1975 1167604 PMC354464

[27] Ferron F, Li Z, Danek EI, Luo D, Wong Y, Coutard B, Lantez V, Charrel R, Canard B, Walz T, Lescar J. 2011. The hexamer structure of Rift Valley fever virus nucleoprotein suggests a mechanism for its assembly into ribonucleoprotein complexes. PLoS Pathog 7:e1002030. doi:10.1371/journal.ppat.1002030 21589902 PMC3093367

[28] Malet H, Williams HM, Cusack S, Rosenthal M. 2023. The mechanism of genome replication and transcription in bunyaviruses. PLoS Pathog 19:e1011060. doi:10.1371/journal.ppat.1011060 36634042 PMC9836281

[29] Mastronarde DN. 2005. Automated electron microscope tomography using robust prediction of specimen movements. J Struct Biol 152:36–51. doi:10.1016/j.jsb.2005.07.007 16182563

[30] Punjani A, Rubinstein JL, Fleet DJ, Brubaker MA. 2017. cryoSPARC: algorithms for rapid unsupervised cryo-EM structure determination. Nat Methods 14:290–296. doi:10.1038/nmeth.4169 28165473

[31] Emsley P, Lohkamp B, Scott WG, Cowtan K. 2010. Features and development of Coot. Acta Crystallogr D Biol Crystallogr 66:486–501. doi:10.1107/S0907444910007493 20383002 PMC2852313

[32] Afonine PV, Poon BK, Read RJ, Sobolev OV, Terwilliger TC, Urzhumtsev A, Adams PD. 2018. Real-space refinement in PHENIX for cryo-EM and crystallography. Acta Crystallogr D Struct Biol 74:531–544. doi:10.1107/S2059798318006551 29872004 PMC6096492

[33] Williams CJ, Headd JJ, Moriarty NW, Prisant MG, Videau LL, Deis LN, Verma V, Keedy DA, Hintze BJ, Chen VB, Jain S, Lewis SM, Arendall WB III, Snoeyink J, Adams PD, Lovell SC, Richardson JS, Richardson DC. 2018. MolProbity: More and better reference data for improved all‐atom structure validation. Protein Sci 27:293–315. doi:10.1002/pro.3330 29067766 PMC5734394

[34] Ren F, Zhou M, Deng F, Wang H, Ning YJ. 2020. Combinatorial minigenome systems for emerging banyangviruses reveal viral reassortment potential and importance of a protruding nucleotide in genome “Panhandle” for promoter activity and reassortment. Front Microbiol 11:599. doi:10.3389/fmicb.2020.00599 32322247 PMC7156889

[35] Mo Q, Xu Z, Deng F, Wang H, Ning YJ. 2020. Host restriction of emerging high-pathogenic bunyaviruses via MOV10 by targeting viral nucleoprotein and blocking ribonucleoprotein assembly. PLoS Pathog 16:e1009129. doi:10.1371/journal.ppat.1009129 33284835 PMC7746268

