# Supplementary material for: Structural insight into RNA encapsidation by the severe fever with thrombocytopenia syndrome virus nucleocapsid protein
Source: mBio. 2025 Oct 31;16(12):e02381-25. doi: 10.1128/mbio.02381-25 (PMC12691673; doi:10.1128/mbio.02381-25)
Supplement: Supplemental material — Fig. S1-S7 and Table S1. [file mbio.02381-25-s0001.pdf]

Supplemental information

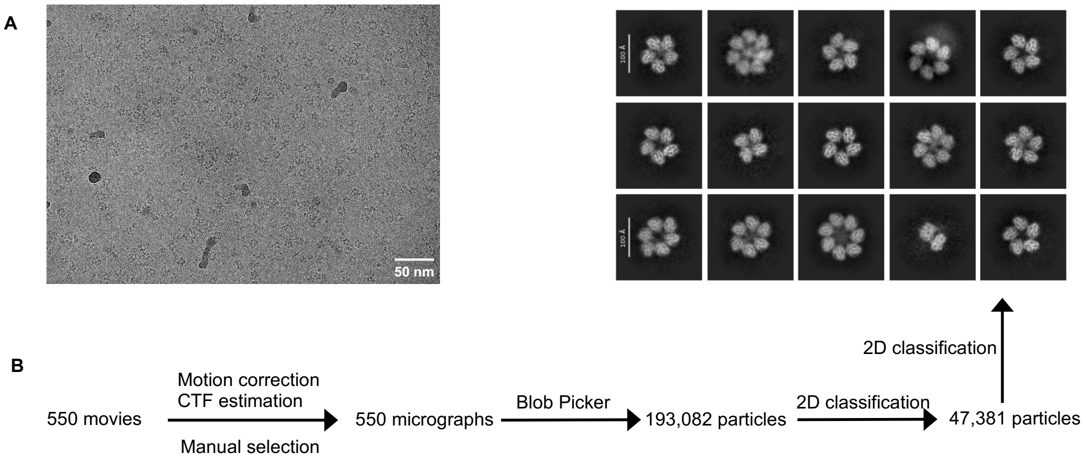

Figure S1. Cryo-EM data processing of the SFTSV NP-RNA complex. (A) Representative cryo-EM image of the SFTSV NP-RNA complex. (B) Flowchart of cryo-EM data processing.

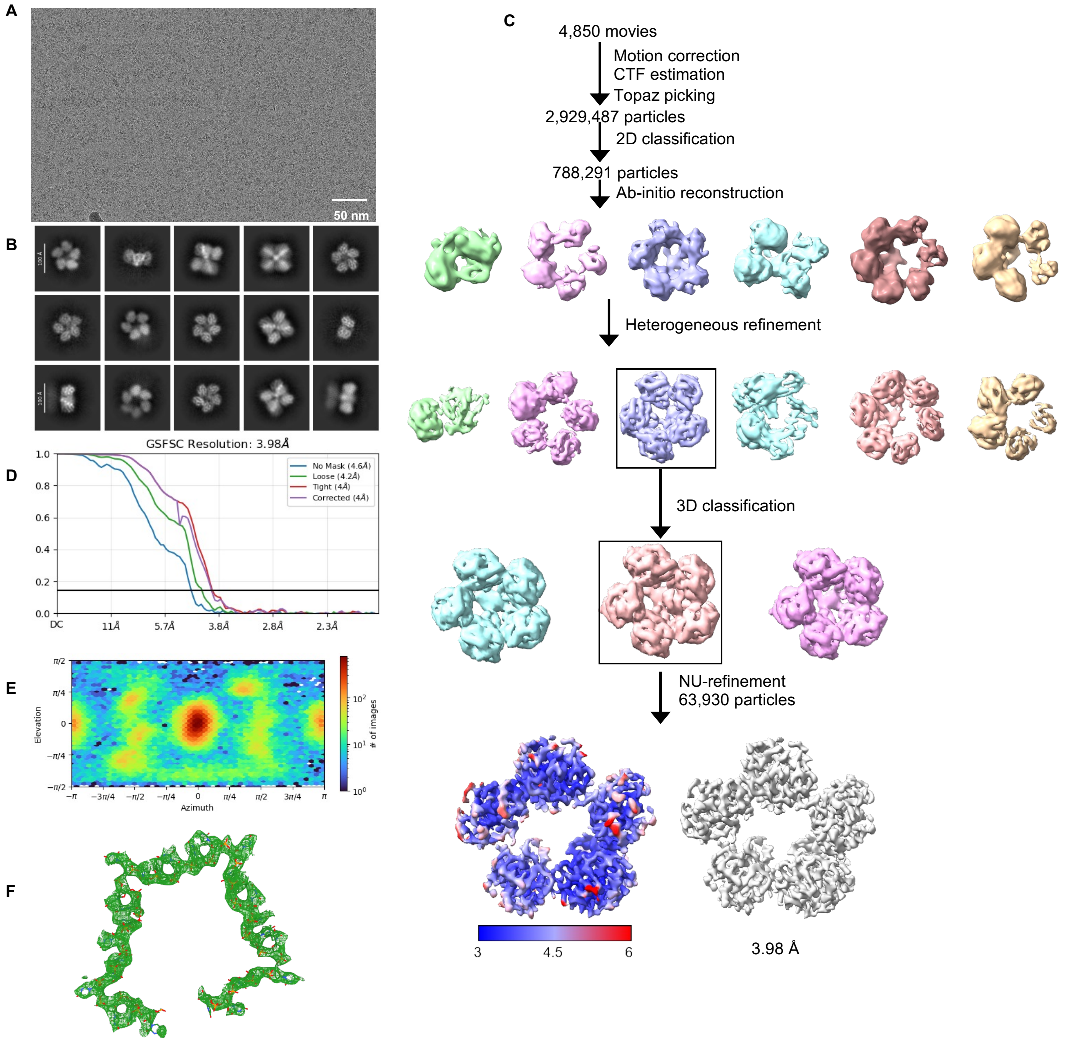

Figure S2. Cryo-EM reconstruction of the PEGylated SFTSV NP-RNA complex. (A) Representative cryo-EM image of the PEGylated SFTSV NP-RNA complex. (B) Representative 2D classification averages of the PEGylated SFTSV NP-RNA complex. (C) Flowchart of cryo-EM data processing. (D) Fourier shell correlation (FSC) curve was calculated using two independent half maps, and resolution was estimated using the FSC=0.143 cutoff. (E) Euler distribution of the refined particles. (F) Cryo-EM density of RNA.

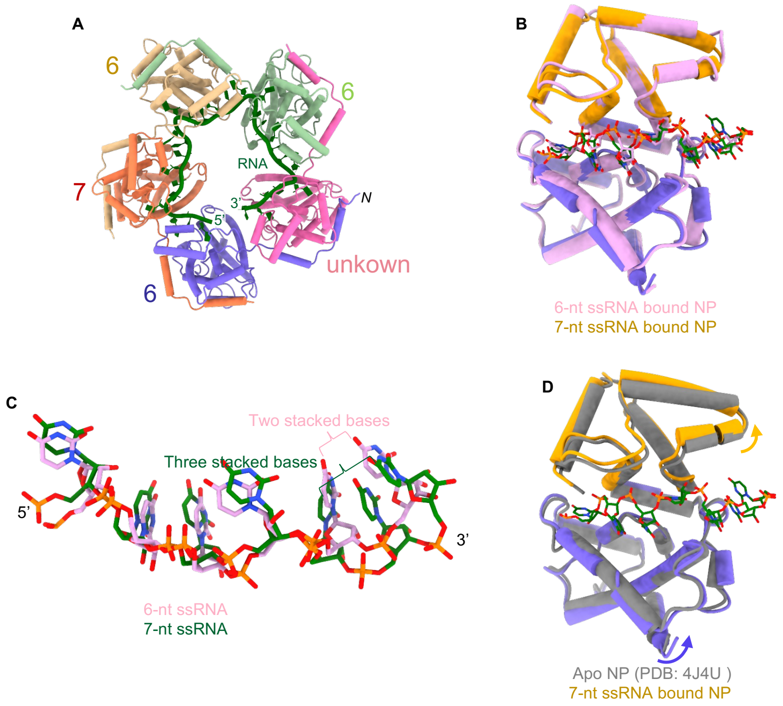

Figure S3. Conformational changes of SFTSV NP. (A) Overall structure of the SFTSV NP-RNA complex, with the number of nucleotides accommodated by each NP protomer indicated. (B) Superimposition of 6-nt ssRNA bound NP and 7-nt ssRNA bound NP. (C) Superimposition of 6-nt ssRNA and 7-nt ssRNA. (D) Superimposition of apo NP protomer and 7-nt ssRNA bound NP.

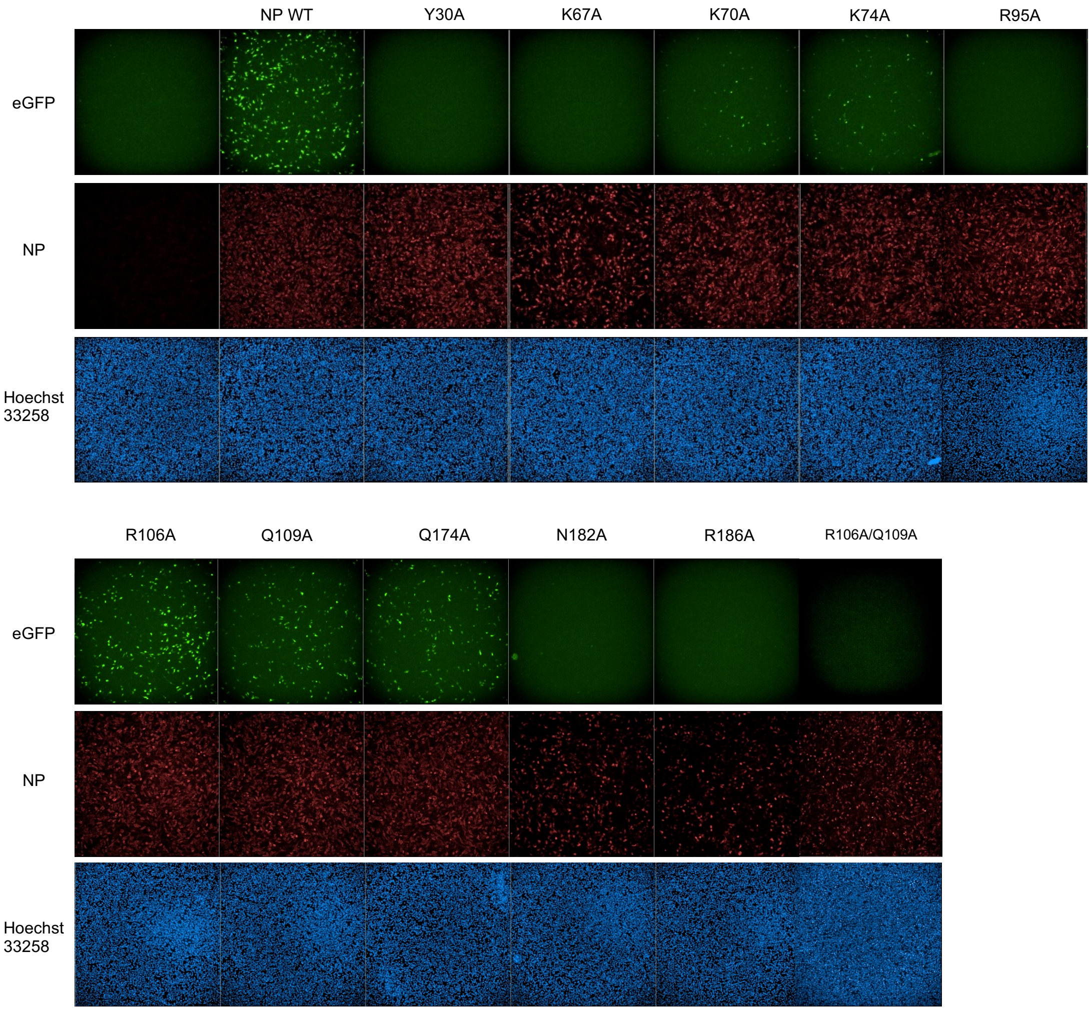

Figure S4. Representative images of minigenome assay. At 48 hours post-transfection, cells were fixed for IFA, and nuclei were stained with Hoechst 33258. eGFP-positive cells were quantified through high-content imaging of nine successive fields per well. Nine fields per well were successively scanned, with one representative field from each group shown.

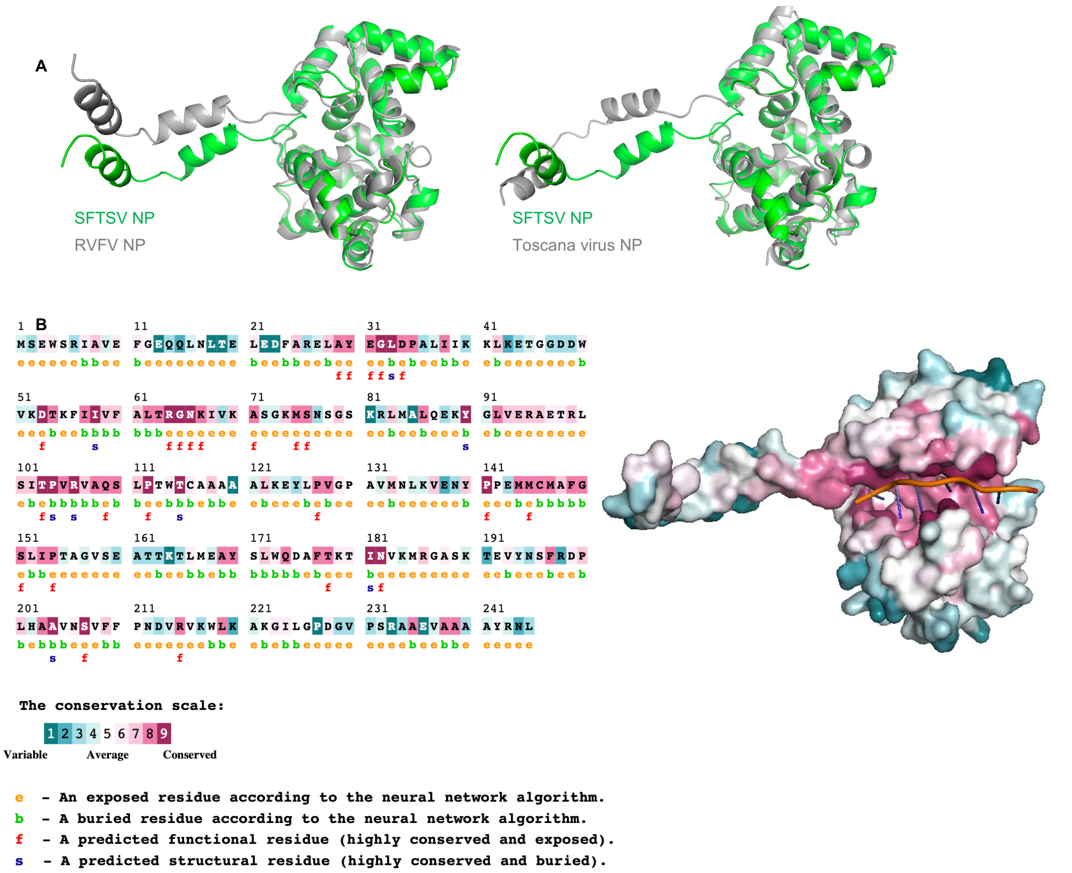

Figure S5. Evolutionary conservation analysis of NP proteins of the *Phenuiviridae* family. (A) Superposition of overall structure of SFTSV NP with RVFV NP (PDB ID: 4H5O) and Toscana virus NP (PDB ID: 4CSF). (B) Alignment of 154 NP protein sequences from the *Phenuiviridae* family shows the residues forming the RNA-binding cleft are highly conserved. Evolutionary conservation was calculated by the ConSurf Server.

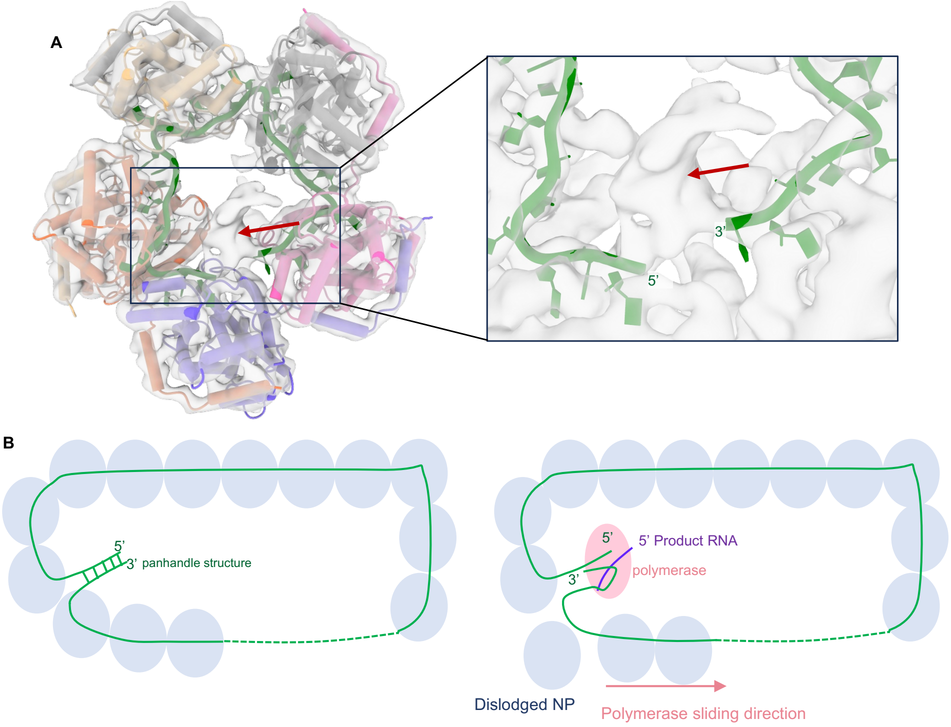

Figure S6. Schematic models of RNP assembly and RNA elongation. (A) Unsharpened cryo-EM map reveals an unmodeled density connects the 5′ and 3′ ends of the RNA. The extra density belonging to the potential RNA duplex is indicated by the red arrow. (B) Schematic model of the SFTSV-RNA complex (left panel). Short complementary sequences at the 5′ and 3′ termini of each viral genomic segment form a base-paired panhandle structure. The viral RNA polymerase recognizes the panhandle structure and initiates RNA synthesis. During elongation, the genomic RNA nearby the replication center becomes transiently uncoated, making it accessible to the RNA polymerase. Meanwhile, the NPs remain held in position by the flexible N-arm. The pink arrow indicates the direction of polymerase sliding (right panel).

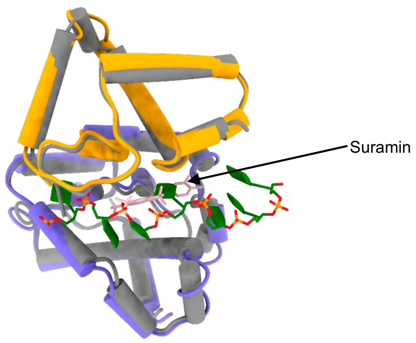

Figure S7. Superimposition of the SFTSV NP-RNA complex with the SFTSV NP-suramin complex. The SFTSV NP-RNA complex follows same color scheme as Fig. S3B. The SFTSV NP-suramin is color grey with suramin colored pink. The PDB ID of the SFTSV NP-suramin complex is 4J4V.

Table S1. Cryo-EM data collection, refinement, and validation statistics

SFTSV NP-RNA complex (EMDB-64333) (PDB 9UMZ)	
**Data collection and processing**	
Magnification	50,000
Voltage (kV)	300
Electron exposure (e–/Å2)	40
Defocus range (μm)	-0.5 to -2.5
Pixel size (Å)	0.95
Symmetry imposed	C1
Initial particle images (no.)	2,929,487
Final particle images (no.)	63,930
Map resolution (Å)<br>FSC threshold	3.98<br>0.143
Map resolution range (Å)	3.0-6.0
**Refinement**	
Initial model used (PDB code)	4J4U
Model resolution (Å)<br>FSC threshold	4.0<br>0.143
Model resolution range (Å)	3.0-6.0
Map sharpening B factor (Å2)	-166.5
Model composition<br>Non-hydrogen atoms<br>Protein residues<br>Nucleotide	 10,018<br>1,224<br>28
B factors (Å2)<br>Protein<br>Nucleotide	 83.2<br>16.3
R.m.s. deviations<br>Bond lengths (Å)<br>Bond angles (°)	 0.002<br>0.569
Validation<br>MolProbity score<br>Clashscore<br>Poor rotamers (%)	 1.57<br>11.33<br>0.00
Ramachandran plot<br>Favored (%)<br>Allowed (%)<br>Disallowed (%)	 98.19<br>1.81<br>0
